# Correction: Mechanism and principle of doping: realizing of silver incorporation in CdS thin film *via* doping concentration effect

**DOI:** 10.1039/d2ra90111k

**Published:** 2022-11-11

**Authors:** A. S. Najm, Abdulwahab Aljuhani, Hasanain Salah Naeem, K. Sopian, Raid A. Ismail, Araa Mebdir Holi, Laith S. Sabri, Asla Abdullah AL-Zahrani, Rashed Taleb Rasheed, Hazim Moria

**Affiliations:** Department of Electrical, Electronics and System, FKAB, Universiti Kebangsaan Malaysia (UKM) 43600 Bangi Selangor Malaysia asmaa.soheil@yahoo.com; Department of Chemical Engineering Technology, Yanbu Industrial College Yanbu Al-Sinaiyah City 41912 Kingdom of Saudi Arabia; Al-Muthanna University Al-Resala Al Muthanna Iraq; Solar Energy Research Institute (SERI), Universiti Kebangsaan Malaysia (UKM) 43600 Bangi Selangor Malaysia; Applied Sciences Department, University of Technology Baghdad Iraq; Department of Physics, College of Education, University of Al-Qadisiyah Al-Diwaniyah Al-Qadisiyah 58002 Iraq; Department of Chemical Engineering, University of Technology Baghdad Iraq; Imam Abdulrahman-bin Fiasal University Eastern Region Dammam Saudi Arabia; Department of Mechanical Engineering Technology, Yanbu Industrial College Yanbu Al-Sinaiyah 41912 Kingdom of Saudi Arabia

## Abstract

Correction for ‘Mechanism and principle of doping: realizing of silver incorporation in CdS thin film *via* doping concentration effect’ by A. S. Najm *et al.*, *RSC Adv.*, 2022, **12**, 29613–29626, https://doi.org/10.1039/D2RA04790J.

The authors regret that the list of affiliations was incorrectly shown in the original manuscript. The corrected list of affiliations is as shown below.

The Royal Society of Chemistry apologises for these errors and any consequent inconvenience to authors and readers.

## Supplementary Material

